# Probiotic significance of *Lactobacillus* strains: a comprehensive review on health impacts, research gaps, and future prospects

**DOI:** 10.1080/19490976.2024.2431643

**Published:** 2024-11-24

**Authors:** Abdul Bari Shah, Aizhamal Baiseitova, Muhammad Zahoor, Ishaq Ahmad, Muhammad Ikram, Allah Bakhsh, Murad Ali Shah, Imdad Ali, Muhammad Idress, Riaz Ullah, Fahd A. Nasr, Mohammed Al-Zharani

**Affiliations:** a Natural Products Research Institute, College of Pharmacy, Seoul National University, Seoul, Republic of Korea; b Division of Applied Life Science (BK21 Four), IALS, Gyeongsang National University, Jinju, Republic of Korea; c Department of Biochemistry, University of Malakand, Chakdara, Pakistan; d Department of Marine Environmental Engineering, Gyeongsang National University, Gyeongsangnam-do, Republic of Korea; e Institute of Pharmaceutical Sciences, Khyber Medical University, Peshawar, Hayatabad, Pakistan; f Department of Oral and Maxillofacial Surgery, University of Texas Health Science Center, San Antonio, TX, USA; g Atta-ur-Rahman School of Applied Biosciences (ASAB), National University of Sciences and Technology (NUST), Islamabad, Pakistan; h Convergence Research Center for Brain Science, Brain Science Institute, Korea Institute of Science and Technology (KIST), Seoul, Republic of Korea; i Centre for Research in Agricultural Genomics (CRAG), CSIC-IRTA-UAB-UB, Campus UAB, Barcelona, Bellaterra, Spain; j Department of Plant Biotechnology, Faculty of Pharmacy, University of Barcelona (UB), Barcelona, Catalonia, Spain; k Department of Pharmacognosy, College of Pharmacy, King Saud University, Riyadh, Saudi Arabia; l Biology Department, College of Science, Imam Mohammad Ibn Saud Islamic University (IMSIU), Riyadh, Saudi Arabia

**Keywords:** *Lactobacillus*, probiotic, gastrointestinal tract, human health

## Abstract

A rising corpus of research has shown the beneficial effects of probiotic *Lactobacilli* on human health, contributing to the growing popularity of these microorganisms in recent decades. The gastrointestinal and urinary tracts are home to these bacteria, which play a vital role in the microbial flora of both humans and animals. The *Lactobacillus* probiotic, i.e, *Lactobacillus plantarum, Lactobacillus paracasei, Lactobacillus acidophilus, Lactobacillus casei, Lactobacillus rhamnosus, Lactobacillus crispatus, Lactobacillus gasseri, Lactobacillus reuteri, and Lactobacillus bulgaricus*, are highly recognized for their remarkable probiotic qualities. The current study aims to highlight the beneficial effects of probiotics in different health conditions, point out the research gap, and highlight the future directives for the safe use of these probiotics in several health issues. Most importantly, we have added the most recent literature related to the characteristics and usage of these probiotics in clinical and pre-clinical settings. Based on the above statement, we believe that this is the first report on the application of probiotics in human diseases. By providing a deeper knowledge of the complex functions these probiotics play in both human and animal health, our analysis will direct future studies and developments in this rapidly developing field.

## 1. Introduction

Gram-positive, non-spore-forming rods called *Lactobacilli* make up a large portion of the normal human bacterial flora. *Lactobacillus* is commonly employed in food manufacturing due to its ability to produce lactic acid and its well-recognized safety. Presently, they are involved in the dietary supplementation of several species, including humans.^1,2^ Their typical anatomical sites are the mouth cavity and the gastrointestinal (GI) tract. The roles of *Lactobacilli* typically encompass the processes of food digestion, nutritional absorption, defense against pathogenic microorganisms, inflammatory modulation, gut flora management, and bacterial infection prevention.^3,4^ Closely associated with luminal microbiota (microbes living in digested food and/or transit stool), these bacteria are known for their intra-site dynamic relationship parietal bacteria (microbes living in mucus layer and/or in intestinal wall). Fascinatingly dynamic and person specific, microbiota composition can be affected by food, probiotic intake, intestinal environment, and other host-dependent events generating some novel bacterial stains. The most common genus in the lactic acid bacteria (LAB) category is *Lactobacilli*, which are involved in the metabolic processes that turns carbohydrates into lactic acid. Based on their metabolic traits, *Lactobacillus* species are often divided into three groups. The primary by-product of the obligatory homofermentative group’s fermentation of carbohydrates is lactic acid (e.g., *Lactobacillus acidophilus* and *Lactobacillus salivarius*). The facultatively heterofermentative group (e.g., *Lactobacillus casei* and *Lactobacillus plantarum*) ferments carbohydrates to produce lactic acid, ethanol/acetic acid, and carbon dioxide as by-products under specific conditions or with specified substrates. Finally, the obligately heterofermentative group continuously ferments carbohydrates to produce carbon dioxide, lactic acid, and ethanol/acetic acid as byproducts (e.g. *Lactobacillus reuteri* and *Lactobacillus. fermentum)*.^5,6^

**Figure 1.**
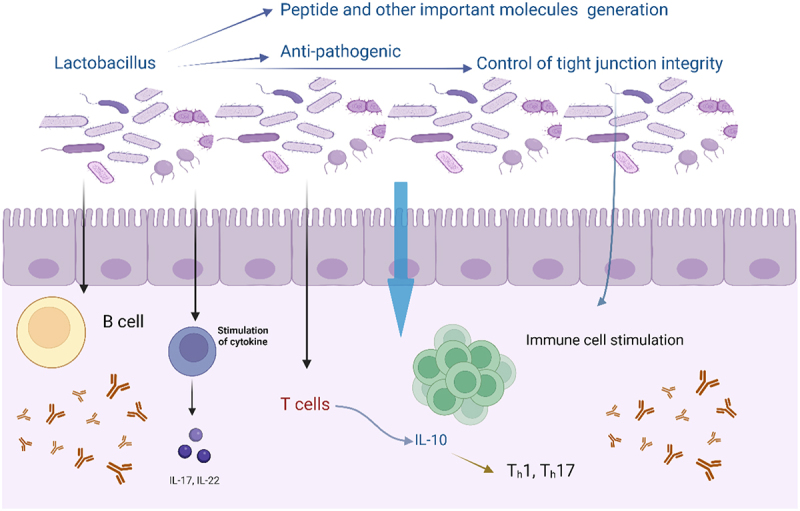
Beneficial role of lactobacillus in intestines, generating beneficial compounds responsible for anti-pathogenic activities and regulation of immune cells, acting as a regulator of tight junction.

**Figure 2.**
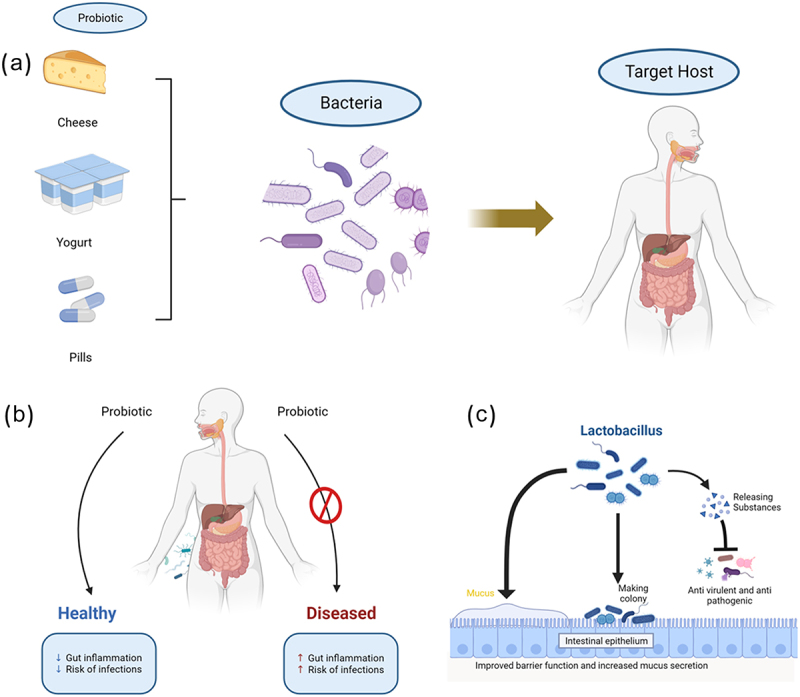
Illustration of probiotic to target host. (a) Sources of probiotic to target area (b) Role of probiotic between healthy and disease person, relating to many other disorders, here we showed the main one (c) Special example of *Lactobacillus*, mechanism of action in intestine.

Over the last twenty years, there has been a notable surge in the interest in probiotic microorganisms, which have successfully demonstrated their beneficial effects on human health and well-being.^7^ It has been shown that these probiotics can improve food quality, shelf life, microbiological safety, and biopreservation. Probiotic foods are dietary supplements that are widely consumed worldwide because of their nutritional worth and potential for the treatment of a variety of human illnesses. For instance, Zhang et al. showed that constipation which is a common gastrointestinal symptom and is related to many other disorders in the human body has a negative impact on life. Different bacteria like *Bifidobacterium* and *Lactobacillus* have been demonstrated to have favorable results in comparison to the modern pharmaceuticals which has adverse effects and related with costly prices.^8^ Some of the bacteria from LAB have a vital function in the generation, intake, and detection of several neurotransmitters. Additionally, it impacts the host’s synthesis, which in turn affects the movement of the digestive system. It regulates the equilibrium of excitatory and inhibitory neurotransmitters involved in gastrointestinal motility, leading to an elevation in motor neuron activity and a reduction in inhibitory neuron activity, ultimately enhancing colonic motility (Specifically, *Pediococcus acidilactici* and *Limosilactobacillus pentosus* increase the levels of serum MTL, Gas, and SP while decreasing the levels of ET, SS, and VIP).^8^ Probiotic effects, however, are still up for contention and need more investigation through long-term human trials. Probiotics have been shown to improve human health through nutrition, although there are still reservations about their use despite the available data. Probiotics can help maintaining the balance of T-cell subsets by promoting the transformation of Th2 to Th1 in particular allergies and Th2-mediated inflammatory diseases (Figure 1). This, in turn, diminishes allergy symptoms. Analogous results have been shown in mouse models of allergies, demonstrating the capacity to improve allergic asthma and atopic dermatitis by promoting the development of Th1 cells while suppressing the responses of Th-2 and Th-17 cells.^9^ An illustration from source to target host of probiotic along with health and disease conditions, also mechanism of action of *Lactobacillus* in intestine, is shown in Figure 2. In the current review we have mainly focused on the most updated studies on *Lactobacillus* in terms of its probiotic properties and its health benefits, and future perspectives/challenges.

## 2. Methodology

The relevant information regarding the *Lactobacillus*, including its probiotic characteristics and health advantages, was collected from published articles. Research publications on probiotic bacteria were located through scientific resources, such as PubMed, Web of Science, Google Scholars, and SciFinder. The selection and evaluation of papers for relevancy were based on titles, abstracts, and keywords. The keywords used was *Lactobacillus* probiotic, *Lactobacillus plantarum, Lactobacillus paracasei, Lactobacillus acidophilus, Lactobacillus casei, Lactobacillus rhamnosus, Lactobacillus crispatus, Lactobacillus gasseri, Lactobacillus reuteri, Lactobacillus bulgaricus* as a probiotic, also irrespective of timeframe but relevant articles were chosen. The revised nomenclature for each species has been incorporated into each title for clarity. The figures were created using Biorender.com.

## 3. Different species of *Lactobacillus* as a probiotic

**Figure 3.**
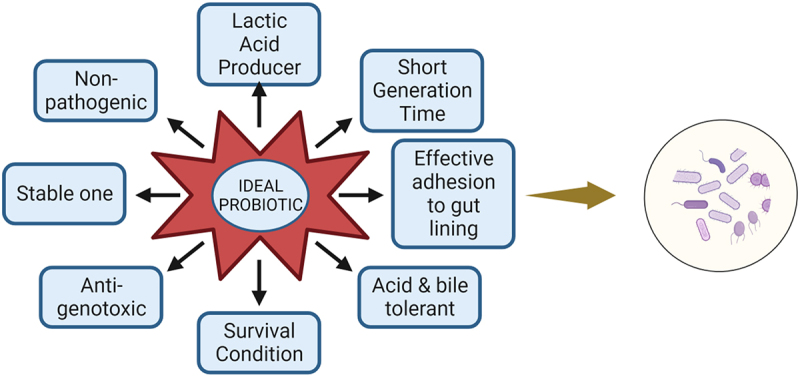
Ideal properties for any bacteria to become probiotic.

**Figure 4.**
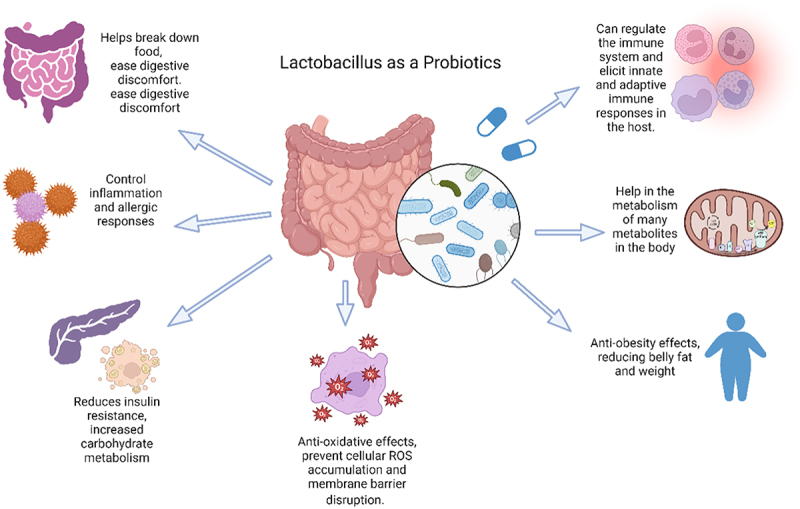
*Lactobacillus* probiotics have a wide variety of positive impacts on the human body, which contribute to some of the health benefits they offer. Its primary purpose is to enhance the functioning of the body as well as the metabolic processes of the body. Most of the portion has been highlighted here.

In last decades probiotics have gained a tremendous interest in the prevention and management of different health issues, and a mounting data has significantly highlighted the mechanisms and effects in these diseased conditions.^5^ Kerry et al. reported the current *Lactobacillus* probiotic are *Lactobacillus plantarum, Lactobacillus paracasei, Lactobacillus acidophilus, Lactobacillus casei, Lactobacillus rhamnosus, Lactobacillus crispatus, Lactobacillus gasseri, Lactobacillus reuteri, Lactobacillus bulgaricus*. ^10^ The probiotic guidelines published by the FAO and WHO may provide a common benchmark for evaluating probiotic content in food, so enabling the verification of health claims (Figure 3). These recommendations require the following actions: identification of the strain(s), functional characterization of the strain(s) for safety and probiotic qualities, human study validation of health benefits, and truthful, non-misleading labeling of efficacy claims and content for the duration of the shelf life.^11^ The health benefits of *Lactobacillus* probiotic are illustrated in Figure 4.

### 3.1. *Lactobacillus plantarum* (*Lactiplantibacillus plantarum*)

The U.S. Food and Drug Administration classifies species of the *Lactobacillus* genus as “generally regarded as safe (GRAS)” due to their long history of safe use in fermented foods and their presence in the typical human intestine and urogenital microbiota.^12^ *Lactobacillus plantarum* (*L. plantarum*), a heterofermentative bacterium, metabolizes both pentose and hexose sugars to produce lactic acid, carbon dioxide, and either acetate or ethanol. *L. plantarum* is a highly promising strain of probiotics primarily present in a wide range of fermented food products. The items mentioned are Pickles, Sauerkraut, Korean Kimchi, Brined Olives, Sourdough bread, Nigerian Ogi, and various other fermented fruits and vegetables. It is also present in specific varieties of cheese, fermented sausages, and stockfish. *L. plantarum* has a long history of being used in the fermentation of dairy, meat, and vegetables. It is one of the most often found *Lactobacillus* species and has been recognized as a food for a long time^12–14^ *L. plantarum* is used in the fermentation of dairy products including cheese and Kefir, pickled vegetables, fermented meat products, and a variety of drinks.^15,16^ Because of *L. plantarum* health claims, various probiotic formulations have been created, and its antibacterial qualities are intriguing for food safety applications like biopreservation technology.^17^ When soshiho-tang, a well-known traditional herbal medication, was fermented with *L. plantarum* KFRI-144, the proliferation of vascular smooth muscle cells decreased. Utilising this strain to make fermented soshiho-tang boosted the suppression of platelet-derived growth factor-BB-induced proliferation of vascular smooth muscle cells. These inhibitory effects were achieved by reducing Akt phosphorylation rates.^18^

The most current research offers fresh insights into the epigenetic mechanism of bacteria-mediated anti-tumor immunity and justifies the use of indole-3-lactic acid produced from *L. plantarum* in therapeutic approaches to treat colorectal cancer in the future.^19^ A study conducted on college students identified *L. plantarum* JYLP-326 as a potentially beneficial approach for alleviating feelings of exam anxiety. The trial had 60 students with anxiety who were randomly allocated to either a placebo or probiotic group, and 30 students without anxiety who did not receive any interventions. Results indicated that administering JYLP-326 reduced symptoms of anxiety, sadness, and insomnia in test-anxious students. The placebo group had a higher level of genetic diversity in their gut microbiota, but the probiotic group demonstrated a higher level of gut microbial diversity. Furthermore, the study revealed a correlation between anxiety and modified fecal metabolomics, which were then reversed after the administration of JYLP-326 supplementation. Evidence indicates that *L. plantarum* JYLP-326 has the potential to be used as a therapeutic intervention for test anxiety.^20^ *L. plantarum* DP189 has the ability to slow down the neurodegeneration induced by the buildup of α-synuclein in the substantia nigra of mice with Parkinson’s disease through the inhibition of oxidative stress, suppression of proinflammatory responses, and modulation of gut microbiota.^21^ The addition of *Lactobacillus curvatus* HY7601 and *L. plantarum* KY1032 to the diet may influence the composition of the human gut microbiota, potentially resulting in a decrease in obesity. These findings contribute to the increasing body of research suggesting that probiotics may modulate the gut microbiota and consequently show therapeutic potential in combating or controlling obesity.^22^

### 3.2. *Lactobacillus paracasei* (*Lacticaseibacillus paracasei*)

*Lactobacillus paracasei* (*L. paracasei)*, a common lactic acid bacterium species used in the fermentation of dairy products. It is typically found in the oral cavity and gastrointestinal tract of humans, as well as in various fermented foods such as yogurt, vegetables, and milk. It offers both health advantages and enhances the taste and texture.^23,24^ *L. paracasei* cells showed potential probiotic properties in the GIT, acting as an anti-enteropathogenic agent and safe for use. The probiotic freeze-drying technique preserved high cell survivability. The banana powder enriched with this bacterium, stored for 60 days, maintained probiotic survival (107 CFU/g) while excluding non-probiotic growth. No further microorganisms were detached during long-term storage due to the moisture content (2.7–3.8%) and water activity (aw) (0.235–0.363). *L. paracasei* was regarded as a very suitable probiotics-banana rehydrated beverage.^25^ Further more in the context of probiotic properties of this species, It has been shown that consuming fermented milk enhanced with *L. paracasei 33* for 30 days improves the quality of life for people suffering from Allergic Rhinitis. The findings indicate that consuming LP-33-fortified fermented milk for a duration of 30 days can significantly and safely enhance the quality of life for individuals suffering from Allergic Rhinitis, suggesting an alternative therapeutic approach for Allergic Rhinitis.^26^ On the other hand, it is important to point out that the FDA has not yet given its approval to any probiotics that are used in this context, which indicates that additional research is required in the future. For eight weeks, taking *L. paracasei* orally (stored at 4°C before intake) at a daily dosage greater than 10 billion bacteria (1×10^10^ CFU) has been shown to reduce important clinical aspects including nose itching in cases of Allergic Rrhinitis. Furthermore, this treatment resulted in a reduction of the pro-inflammatory mediator IL-5 production.^27^ The probiotic *L. paracasei* K5, derived from dairy products, was used to make cheese that resembles Feta. An immobilized biocatalyst consisting of *L. paracasei* K5 cells and wheat bran prebiotic improved the Feta-type cheese’s acceptability, quality, and aroma. It also increased its shelf life.^28^

In most recent studies, the synergistic effects of the various components of the *L. paracasei* CNCM I-5220-derived postbiotic (LP-PBF) help to preserve gut homeostasis. The findings imply that LP-PBF may find use in the treatment of several diseases that exhibit compromised intestinal barrier performance.^29^ In another recent finding, Kumaree et al. looked at the potential advantages of new probiotic strains in supporting healthy aging as well as their capacity to guard against the toxicity of amyloid β, which is linked to Alzheimer’s disease. It was also looked at how four distinct probiotics (*Lactobacillus salivarius, L. rhamnosus, L. reuteri, and L. paracasei HII01*) affected the *Caenorhabditis elegans* model ability to live longer. The results showed that *L. paracasei* HII01 had the greatest beneficial impact on longevity and showed signs of anti-aging in *C. elegans*. An investigation into the putative DAF-16-mediated mechanism for modifying the lifespan extension mediated by *L. paracasei* HII01 was found through the analysis of qPCR data and research employing mutant variants. Furthermore, the probiotic strains provided protection against the toxicity produced by transgenic *C. elegans* strains that express β-Amyloid (Aβ). Among these strains, *L. paracasei* HII01 displayed the highest degree of protection.^30^

### 3.3. *Lactobacillus acidophilus* (*Lacticaseibacillus acidophilus*)

*Lactobacillus acidophilus (L. acidophilus)* is a rod-shaped, homofermentative, anaerobic, Gram-positive bacteria that was first isolated from baby feces in 1900.^31,32^ It is primarily found in humans, predominantly in the oral cavity, vagina, and gastrointestinal tract. *L. acidophilus* has stronger tolerance to both acid and bile salt than many other probiotics. These features let *L. acidophilus* survive and proliferate in the demanding conditions of the gastrointestinal tract.^33^ Additionally, it can be found in a wide range of fermented foods, including yogurt and fermented milk, amongst others. *L. acidophilus* is a strain that is frequently used in commercial dairy production due to the powerful probiotic effects it offers. It prefers low pH values (below 5.0) and operates best at 37°C, which is recommended for growth. The genome of *L. acidophilus* has previously been established owing to the sequencing of DNA.^34,35^ *L. acidophilus* in baby formula resulted in a decrease in infant blood cholesterol levels from 147 mg/100 ml on the 5th day to 119 mg/100 ml on the 8th day of the study. The reduction in serum cholesterol levels was followed by a noteworthy increase in the quantity of LAB.^36^ To sustain the balance of intestinal flora, *L. acidophilus* can produce metabolites and reduce the gut’s pH. Some pathogenic bacteria generate enzymes that catalyze the conversion of carcinogenic precursors into carcinogens. The enzymes comprise nitro reductase, azo reductase, and β-glucosidase. *L. acidophilus* can inhibit the proliferation of pathogenic microorganisms, reduce enzyme production, and obstruct enzyme activity.^33,37^ *L. acidophilus* has been shown in studies to provide a variety of probiotic benefits on humans, including as decreasing cholesterol, promoting immunological response, assisting in lactose digestion, and serving as a barrier against infections. The presence of *L. acidophilus* at concentrations between 10^5^ and 10^6^ CFU per milliliter is required to see these effects.^33,38^ Most recently, Jeon et al. isolated and characterized a feruloyl esterase from *L. acidophilus*, a powerful antioxidant used in numerous industries. The outcomes showed that feruloyl esterase reacts with ethyl ferulate and can be utilized to efficiently extract ferulic acid from maize stalks, rice bran and wheat bran.^39^ Treatment with *L. acidophilus* suppressed the increases in TLR2 and TLR4 expression generated by SesE in HT29 cells. Cells that were pretreated exhibited markedly elevated expression of TLR2 and TLR4 compared to cells infected with SesE but not subjected to pretreatment. These findings indicate that *L. acidophilus* has the ability to reduce inflammation and regulate the body natural immunological response to SesE via affecting the expression of TLR2 and TLR4.^40^ Another study showed that *L. acidophilus* LA5 effectively regulated the loss of alveolar bone caused by infection from periodontopathogens. As a result, there were alterations in the composition of the microorganisms in the mouth and digestive tract, suggesting an imbalance in the microbiomes. *L. acidophilus* LA5 reduced the elevated levels of *Enterococaccea*, *Streptoccocaceae*, *Staphylococcaceae*, *Moraxellaceae*, and *Pseudomonadaceae* in the oral microbiome of the periodontitis group. On the other hand, *L. acidophilus* LA5 caused a rise in the superphylum *Patescibacteria* and the family *Saccharamonadaceae* in the intestines of mice that were not infected. These data suggest that *L. acidophilus* LA5 is a potential probiotic for managing periodontitis.^41^

### 3.4. *Lactobacillus casei* (*Lacticaseibacillus casei*)

Gram-positive, rod-shaped, non-sporulating (non-spore-forming), non-motile, anaerobic bacteria are the defining characteristics of the species *Lactobacillus casei* (*L. casei)*. These strains, which are frequently used in the fermentation of foods like cheese and yogurts, have been grown and studied.^1^ *L. casei* is found in cheddar cheese, with *L. casei* and *L. rhamnosus* recognized as the predominant species in cheddar manufactured in Australia and New Zealand. Furthermore, Sicilian green Olives that are naturally fermented also include a major species, *L. casei*. ^42,43^ *L. casei* works to prevent infections caused by *Clostridium difficile* and antibiotic-associated diarrhea (AAD) (CDI).^44^ Oral *L. casei* Zhang can also fix bilateral renal ischemia-reperfusion-induced gut microbial dysbiosis, reduce kidney damage, and slow down its development to chronic kidney disease (CKD).^45,46^

In the most recent exploration, it was found that *L. casei* and *L. reuteri* were able to inhibit TLR4, which in turn stopped pancreatic cancer cells from multiplying, migrating, invading, and inducing macrophages to become M2 polarized. In addition, *L. casei* and *L. reuteri* were able to reduce the growth of pancreatic cancer and improve the polarization of M1 macrophages. According to the findings, *L. casei* and *L. reuteri* reduce the risk of pancreatic cancer by inhibiting TLR4, which in turn promotes the M1 polarization of macrophages and helps to maintain the homeostasis of the gut microbiota.^47^ Hosseinzadeh et al. investigated the possible method by which *L. casei* condition medium mediates its apoptotic impact in colorectal cancer cells through downregulation of miR-21.^48^ Also, the antagonist effect of *L. casei* and *L. rhamnosus* was investigated against the biofilm of *Staphylococcus aureus*, which displayed a potent antibiofilm effect.^49^

An industrial, commercial, and applied health potential makes *L. casei, L. paracasei, and L. rhamnosus*, has received the most attention from researchers.^50^ The strain *L. casei*-01 has been persistently employed as a probiotic culture in dairy products historically. Conversely, it has also emerged as an approach for obtaining dairy-free probiotic. *L. casei*-01 has recently been used into whey-protein or polysaccharide-based edible coatings applied to meat, baked items, and fruits.^51^

### 3.5. *Lactobacillus rhamnosus* (*Lacticaseibacillus rhamnosus*)

Originally identified as a subspecies of *Lactobacillus rhamnosus*, *Lacticaseibacillus rhamnosus* was commonly known as *Lactobacillus rhamnosus (L. rhamnosus)*. Subsequent genomic research, however, indicated that it was a unique species within the *L. casei* lineage, belonging with *L. paracasei* and *L. zeae*. ^1,52,53^ When it comes to protecting kids from diarrhea caused by rotavirus, *Lacticaseibacillus rhamnosus* GG works well. It has proven effective in treating and preventing various types of diarrhea in people of all ages, from toddlers to adults. The European recommendations have recently endorsed *L. rhamnosus* GG, indicating that it has demonstrated potential in preventing hospital-acquired diarrhea and diarrhea linked to antibiotic use.^54–58^ Mathipa-Mdakane et al. reviewed up to 2022 that *L. rhamnosus* presents a promising candidate for probiotic engineering to develop novel strains with enhanced pathogen-specific inhibition and health benefits.^59^

Recently, it was found that administering *L. rhamnosus* GG to ovariectomized rats resulted in enhanced bone microarchitecture, biomechanics, and expression levels of CTX-I, PINP, Ca, and RANKL. Additionally, it facilitated the formation of new bone tissue, perhaps providing relief from osteoporosis. The administration of *L. rhamnosus* GG altered the equilibrium between Th17 and Treg cells and mitigated inflammation and damage to the intestinal barrier caused by estrogen deprivation. In summary, *L. rhamnosus* GG improved the condition of osteoporosis caused by estrogen shortage by controlling the gut microbiome and intestinal barrier, as well as promoting a balance between Th17 and Treg cells in both the gut and bone.^60^ *L. rhamnosus* suppressed the activation of the NF-κB/c-Fos/NFATc1 pathway, which in turn prevented osteoclast development. Moreover, it prevented the breakdown of the alveolar bone, offering a fresh approach to the application of probiotics in the management of periodontitis.^61^ Furthermore, by concurrently decreasing Th1 cells, *L. rhamnosus* can effectively block CD8^+^T cell-mediated inflammation, hence improving the efficacy of rheumatoid arthritis treatment. Ninety-nine individuals with rheumatoid arthritis took part in this investigation.^62^

### 3.6. Lactobacillus crispatus

One common rod-shaped member of the *Lactobacillus* genus that is useful for producing hydrogen peroxide (H_2_O_2_) is *Lactobacillus crispatus (L. crispatus)*. It can be detected in the gastrointestinal system of vertebrates as well as in the vaginal environment through vaginal discharge.^63^ *L. crispatus L1* has been shown to have potential as a vaginal probiotic by Donnarumma et al. that emphasized the significance of fermentation processes in order to obtain higher quantities of viable cells.^64^ Czaja et al. conducted a Phase I Trial involving a *L. crispatus* vaginal suppository aimed at preventing recurrent urinary tract infections in women. Their findings suggest that *L. crispatus* CTV-05 can be administered as a vaginal suppository with minimal side effects in healthy women with a history of recurrent UTI.^65^ Furthermore, the probiotic strain CTV-05 is employed for both premenopausal and postmenopausal women dealing with recurrent urinary tract infections. It is undergoing assessment, particularly for its effectiveness in preventing and treating bacterial vaginosis, a condition marked by the deficiency of essential *Lactobacillus* flora crucial for host protection against infections.^66–68^

In recent studies, *L. crispatus* plays a key role in bacterial vaginosis, a prevalent disorder affecting one-third of women globally. Electrospun fibers loaded with *L. crispatus* demonstrate the ability to generate viable and metabolically active bacteria, effectively eliminating Gardnerella. The study explores the potential of *L. crispatus* loaded fibers as a biocompatible platform for treating bacterial vaginosis. These fibers, composed of FDA approved polymers such as PLGA and PEO, offer sustained release of probiotics, lactic acid, and hydrogen peroxide. It’s important to note that this proof of concept study has limitations, as it focuses on evaluating one probiotic strain against a single anaerobic bacterial strain.^69^ *L. crispatus* has been shown in randomized clinical trials to prevent and treat intrauterine adhesion, suppress endometrial fibrosis, and restore the vaginal microbiota following intrauterine surgery. In addition, it is a novel investigation into the use of vaginal probiotics in the treatment of gynecological disorders.^70^

### 3.7. *Lactobacillus gasseri* (*Ligilactobacillus gasseri*)

The US Food and Drug Administration has designated *Lactobacillus gasseri (L. gasseri)* as Generally Recognised as Safe (GRAS). It is facultative anaerobic lactic acid bacteria.^71^ This microbe is common in the female reproductive system, gastrointestinal system, and oral cavity of humans. Among the many probiotic qualities that *L. gasseri* demonstrates are the control of intestinal flora, anti-inflammatory and antibacterial actions, preservation of the equilibrium of female vaginal flora, and uric acid reduction. It is positioned as a prospective probiotic candidate by these qualities.^72,73^ *L. gasseri* HHuMIN D serves as a secure and bioactive lactobacterial component, suitable as a food ingredient, starter culture, or probiotic microorganism for promoting human oral health.^74^ *L. gasseri* generates Gassericin A, which is a bacteriocin, a proteinaceous or peptidic toxin produced by bacteria to impede the growth of similar or closely related bacterial strains.^75,76^

Recently, it is reported that *L. gasseri* LA39 enhances the biotransformation of intestinal secondary bile acids and promotes the hepatic generation of primary bile acids. The results imply that by controlling bile acid metabolism, *L. gasseri* LA39 provides a crucial role in the gut-liver axis.^77^ It has been shown by Gao et al. that *L. gasseri* LGV03, which was isolated from the cervico-vagina of women who had cleared human papillomavirus, can control innate immune responses in the epithelium. Furthermore, it was discovered that this strain prevented human cervical cancer cells that tested positive for human papillomavirus from growing.^78^ Also, Insulin resistance and liver damage brought on by type 2 diabetes were reduced by the *L. gasseri* CKCC1913-mediated regulation of the gut-liver axis, highlighting *L. gasseri* CKCC1913 and its potential impact on metabolic health highlight the favorable potential of probiotics as a therapeutic treatment for diabetes.^79^

### 3.8. *Lactobacillus reuteri* (*Limosilactobacillus reuteri*)

*Lactobacillus reuteri (L. reuteri)* is an extensively researched probiotic bacterium colonizing a large number of mammals. In humans, *L. reuteri* is present in diverse bodily locations such as the gastrointestinal tract, urinary tract, skin, and breast milk. The prevalence of *L. reuteri* varies among individuals.^80^ *L. reuteri* serves as a preventive measure for this ailment, children who receive it when in good health are less prone to developing diarrhea. In terms of safeguarding against intestinal infections, comparative studies indicate that *L. reuteri* exhibits greater efficacy than other probiotics.^81^ Severe enterocolitis is caused by high dosages of the chemotherapy medication methotrexate. *L. reuteri* dramatically reduces the signs and symptoms of methotrexate-induced enterocolitis in rats, including bacterial translocation.^82^ *L. reuteri* has demonstrated the potential to enhance dental health by effectively eliminating *Streptococcus mutans*, a bacterium associated with tooth decay. *L. reuteri* was the only probiotic bacteria that showed the ability to inhibit *Streptococcus mutans* out of all those that were examined. A different investigation established that *L. reuteri* does not negatively impact teeth prior to human testing. Clinical experiments later showed that those whose diet supplemented with *L. reuteri* had a significantly lower amount of *Streptococcus mutans* in their oral microbiome.^83^

A study reveals a significant interaction between CD8 T cells and indole-3-aldehyde, an aryl hydrocarbon receptor agonist produced by probiotics, in the tumor microenvironment. This interaction enhances anticancer immunity and increases the effectiveness of immune checkpoint inhibitors. *L. reuteri*, a probiotic, can colonize and translocate to melanoma, enhancing its anti-tumor action. However, this tumor translocation capability is not exclusive to *L. reuteri* and may also be seen in other commensal bacteria. Dietary tryptophan can also elicit similar anti-tumor action by metabolizing tryptophan into indole-3-aldehyde, stimulating CD8 T lymphocytes to produce interferon-γ. This process enhances the effectiveness of immune checkpoint inhibitors and prolongs life in advanced melanoma patients.^84^ *L. reuteri* restored the intestinal barrier, reduced pulmonary edoema, slowed the inflammatory response, and altered the gut microbiota in acute lung injury mice. This offers new insights into the clinical management of acute lung injury.^85^

### 3.9. *Lactobacillus bulgaricus* (*Lactobacillus delbrueckii subsp. bulgaricus*)

Gram-positive*Lactobacillus bulgaricus (Lactobacillus delbrueckii ssp. bulgaricus), (L. bulgaricus)* is a rod that frequently appears lengthy and filamentous. It doesn’t move and doesn’t make spores. It is nonpathogenic as well. This bacterium is classified as acidophilic or aciduric and prefers low pH environments, usually between 5.4 and 4.6. Moreover, it is anaerobic.^86,87^ Using the azoxymethane/dextran sodium sulfate paradigm, Silveira et al. showed that *L. bulgaricus* reduces colitis-associatedcancer by adversely regulating intestinal inflammation.^88^ Furthermore, encapsulating *L. bulgaricus* in alginate – milk microspheres serve as an effective protective measure against harsh simulated gastrointestinalconditions.^89^

In recent finding, derivatives of proteins and exopolysaccharides from *L. bulgaricus* were extracted, characterized, and for the first time used in the production of novel self-crosslinking 3D printed alginate/hyaluronic acid hydrogels, as high-value functional biomaterials with therapeutic potentials in regenerative medicine applications.^90^ The two primary species utilized in the manufacture of yogurt are *Streptococcus thermophilus* and *L. bulgaricus*. According to Xue et al. the glutathione produced by *S. thermophilus* during cocultivation successfully increased *L. bulgaricus* activity and markedly raised the fermented milk quality.^91^ Guo et al. first employed low acyl gellan gum to immobilize *L. bulgaricus* T15. It was employed in the synthesis of D-lactic acid from non-detoxified maize stover hydrolyzate, demonstrating exceptional efficacy as a cell immobilization medium with significant potential for application in the straw biorefinery sector.^92^Table 1 show the probiotic properties of these nine *Lactobacillus* species.

**Table 1.** *Lactobacillus* and their probiotic properties, special focus on the nine species that were discussed in the review.

Bacteria	Probiotic Properties and Clinical Manifestation	Ref
*Lactobacillus plantarum*	Survive at pH 2, highest hydrophobicity (79.13%), represented the deconjugation of bile salts, highest cholesterol reduction (59%). Twenty-five strains derived from fermented foods, safe for consumers, can tolerate the simulated gastrointestinal fluids. Acid-bile tolerance, antibiotic sensitivity, auto-aggregation, co-aggregation, bacterial adhesion to hydrocarbons were confirmed from two strains Randomized trails: meta-analysis showed, it can enhance host immunity by regulating both pro-inflammatory and anti-inflammatory cytokines.	^142–145^
*Lactobacillus paracasei*	*L. rhamnosus* IMC 501 and *L. paracasei* IMC 502 can be used as probiotics in functional foods due to its high adhesion ability. Strain L1 supplementation promote growth and upgrade the intestinal microflora in chicken Use of *l. casei* and *l. paracasei* in clinical trials for the enhancement of human health has been done in many studied and considered as safe.	^146–148^
*Lactobacillus acidophilus*	AD125 strain had higher gastrointestinal tolerance, auto aggregation percentage (26.51 ± 0.71%), and coaggregation percentage (23.97 ± 0.44%) with *E. coli* O157:H7, high surface hydrophobicity of toluene and xylene (83.59 ± 2.54% and 93.45 ± 1.24%) Clinical trials: *L. acidophilus* NCFM and *B. lactis* Bi-07 improve symptoms of bloating in patients with functional bowel disorders.	^149,150^
*Lactobacillus casei*	*L. casei* MYSRD 108 and *L. plantarum* MYSRD 71 strains exhibited strong survival and antagonistic activities for probiotic application in the gastrointestinal tract against *Salmonella paratyphi* biofilm. Yogurt with *L. casei* Zhang remained viable (1.0 × 10^9^ cfu/mL) after 28 days, suggesting its potential for functional foods and health products.	^151,152^
*Lactobacillus rhamnosus*	*L*. *rhamnosus* 4B15) showed high tolerance to acid and bile salts, and ability to adhere to the intestine. Have an impact on immune health by modulating pro-inflammatory cytokines. Randamozed clinical trials: Supplementing with the probiotic *L. rhamnosus* GG enhanced cognitive performance in middle-aged and older individuals who had cognitive impairment.	^153,154^
*Lactobacillus crispatus*	*L.crispatus 2029*, producing H_2_O_2_, exhibits broad antagonistic activity, enhancing colonization resistance against agents associated with urinary tract infections, bacterial vaginosis, and vulvovaginal candidiasis. *L. crispatus* exhibited resistance to methicillin, metronidazole, oxacillin, and sulfamethoxazole + trimethoprim, but the bacteria displayed sensitivity to examine the other antibiotics. Clinical trial: The strain *L. crispatus* FSCDJY67L3 has potential clinical applications as a supplement for treating *H. pylori* infections.	^155–157^
*Lactobacillus gasseri*	*L. gasseri* LGZ1029 displays favourable gastrointestinal tolerance, bacteriostatic capability, and antioxidant activity, showcasing excellent probiotic traits, suggesting highly promising probiotic candidate. Display great capability of exopolysaccharide production, and tolerance to acid and bile salt. Clinical trial: *L. gasseri* BNR17 has the potential to improve diarrhea-IBS as a probiotic.	^158–160^
*Lactobacillus reuteri*	Withstanding low pH and enzyme-rich conditions, adhering to epithelium for host-probiotic interaction, and competing with pathogenic microorganisms. Randomized control trial: Consuming probiotic *L. reuteri*-lozenges is an effective method to enhance and sustain periodontal health when personal oral hygiene becomes less effective.	^85, 161^
*Lactobacillus bulgaricus*	Strains LB1, LB2 and LD, showed 85.59% survival rates at pH 3.0 of the acid and 96.73-64.51% at 0.1%-0.3% bile salt concentration, 61.88% cell surface hydrophobicity. *L. bulgaricus* KLDS 1.0207 as a capable probiotic candidate having antimicrobial, anti-inflammatory, acid, and bile tolerant and lipid-regulating properties. Randomized control trial: Consuming yoghurt that has been fermented with *L. delbrueckii* ssp. bulgaricus OLL1073R-1 improves the subjective psychological quality of life for women healthcare professionals.	^162–164^

## 4. The historical evolution of traditional fermented beverages

Fermented foods, a traditional food preservation method, are gaining popularity due to the health benefits of a balanced gut microbiota. Fermentation, which uses microbes like *Lactobacillus*, transforms raw ingredients into savory, nutrient-dense meals, attracting consumers due to their perceived health benefits.^93,94^ Especially *Lactobacillus* strains, which are well-known for enhancing gut flora, probiotics abound in fermented foods including kimchi, sauerkraut, koumiss, yogurt, kurut, cheese, Kefir and kombucha. Here are some traditional food sources of *Lactobacillus*:

- Yoghurt: One of the most well-known sources of *Lactobacillus*. Yoghurt with living, active cultures is what you want because it contains good bacteria, including various *Lactobacillus* strains.^95^
- Koumiss: A dairy product made from fermented mare’s milk, it is renowned throughout Asia, Russia, and many other countries. It is consumed in both solid and liquid form, serving as both a source of nourishment and an alcoholic drink.^96^
- Kurut: The product is derived from raw cow milk that has not been pasteurized, and it undergoes spontaneous fermentation by microorganisms present in the air.^97^
- Fermented Cheese: *Lactobacillus* can be present in some cheese varieties, particularly those that go through a fermentation process. Cheddar, Swiss, and Gouda are a few examples.^98^
- Kefir: A fermented milk beverage prepared using Kefir grains. Numerous probiotic microorganisms are present in it, including *Lactobacillus* species.^99^
- Sauerkraut: Fermented cabbage, rich in several strains of *Lactobacillus*. In many cultures, it’s a common side dish and condiment.^100^
- Kimchi: A traditional Korean meal made with fermented vegetables, typically radishes and cabbage, and spiced with ginger, garlic, and chili peppers. Kimchi is a source of *Lactobacillus* bacteria.^101,102^
- Miso: A traditional Japanese condiment made by combining koji (a type of fungus) and salt to ferment soybeans. One of the helpful bacteria that aids in fermentation is *Lactobacillus*. ^103^
- Pickles: *Lactobacillus* is abundant in fermented cucumbers. Instead of using vinegar, make sure they are naturally fermented in brine.^104^
- Buttermilk: The liquid that remains after churning butter, known as traditional buttermilk, is frequently fermented and contains *Lactobacillus* bacteria.^105^
- Tempeh: A traditional Indonesian soy product made by fermenting soybeans. The fermentation is mainly process by *Lactobacillus* bacteria.^106^
- Sourdough Bread: Traditional sourdough bread is leavened through a natural fermentation process that involves *Lactobacillus*, among other microorganisms.^107^

With a rich and centuries-long history, Kefir is a fermented milk beverage with a distinct effervescence and a somewhat acidic taste. Kefir is a fascinating cultural and gastronomic phenomenon, with its origins entwined with traditions and stories. The Caucasus mountains, which lay between Georgia and Russia, are thought to be the birthplace of Kefir. It is said that the Kefir grains were accidentally exposed to the milk that the nomadic tribes in this region carried in leather pouches during their milk-carrying practises. The secret to fermenting milk and making Kefir is Kefir grains. The precise source and discovery of Kefir grains are still mostly unknown. The drink was significant to culture and was seen as a source of endurance and power. Due to the efforts of Russian physicians and scientists, Kefir was introduced to Russia in the late 1800s. The Russian medical community started researching Kefir’s qualities after realizing that it might have health benefits. Russian scientists and health officials became interested in Kefir in the early 20th century after learning about its possible health benefits.^108–112^

*Lactobacillus*, *Streptococcus*, and yeast species such as *Saccharomyces* were found to contribute to the fermentation process.^113^ Kefir has had a global comeback in popularity in recent decades due to growing interest in probiotics and fermented foods for their possible health benefits. These days, it’s easily found at health food stores and supermarkets in a variety of forms, including as conventional milk Kefir, water Kefir, and even nondairy substitutes.^114^ *Lactobacillus acidophilus, Bifidobacterium bifidum, Streptococcus thermophilus, Lactobacillus delbrueckii subsp. bulgaricus, Lactobacillus helveticus, Lactobacillus Kefiranofaciens, Lactococcus lactis*, and *Leuconostoc* species are just a few of the probiotic bacteria found in Kefir products.^115,116^ The Kefir polysaccharide is synthesized in large part by these bacteria. Kefir is now praised for both its distinct flavor and possible health benefits linked to the variety of microorganisms that make up its composition. The Kefir story is an intriguing one in the realm of fermented foods because it shows how cultural customs, myths, and scientific knowledge interact.^113^ There are many other fermented products that are discussed before contain *Lactobacillus* probiotic for fermentation.

## 5. Possible side effects of *Lactobacillus*

*Lactobacillus*, however, is associated with some illnesses, particularly in individuals with weakened immune systems. There have been numerous recorded cases of persons experiencing illnesses caused by *Lactobacillus* species. The patients encompass individuals with AIDS, neutropenia, and individuals who have undergone organ transplants. The most prevalent diseases caused by *Lactobacilli* are localized infections such as abscesses, bacteremia, and endocarditis.^117,118^ Additionally, the risk factors commonly reported in the literature for *Lactobacilli* infections include Diabetes mellitus, preexisting structural heart disease (in cases of infective endocarditis), cancer (particularly leukemia), the utilization of total parenteral nutrition, the administration of broad-spectrum antibiotics, chronic kidney disease, inflammatory bowel disease, pancreatitis, chemotherapy, neutropenia, organ transplantation (especially liver transplantation), and the use of steroids.^119–123^ Following the implementation of the Dietary Supplement Health and Education Act (DSHEA) in 1994, enforced by the US Food and Drug Administration (USFDA), probiotics have attained considerable popularity as dietary supplements in the United States. The more lenient rules under DSHEA have enabled their promotion and over-the-counter distribution in the United States. It is essential to acknowledge that the DSHEA is applicable just inside the United States, and its impact does not reach the international use of probiotics, including *Lactobacillus* species. The function of *Lactobacillus* in global disease management warrants further investigation, since the application of probiotics in certain medical situations may be contraindicated. Moreover, regulatory frameworks beyond the U.S. may enforce more stringent regulations on probiotics, potentially influencing the utilization and perception of both *Lactobacillus* and non-*Lactobacillus* species worldwide.^124–126^

## 6. Research gap, future challenges, and conclusive remarks

The human gut is colonized by a wide variety of probiotic bacteria that actively interact and co-evolve with the host organism. These microorganisms are vital for the digestion and absorption of food, the metabolization of toxic waste products, and the synthesis of important molecules like short-chain fatty acids and amino acids, which are necessary for regular physiological processes. Their presence produces observable health benefits, particularly through improving the host’s microecological balance and positively influencing the gastrointestinal system.^127^ Global sales of health goods bearing the label “probiotic” have increased dramatically since the early 1990s. Simultaneously, “probiotics” have become a hot topic in global research. Numerous disorders have been thoroughly investigated by these microbes, revealing a wide range of potential health impacts, that has been described in each section of different lactobacillus strain previously.^128^ It’s hardly unexpected that the global probiotics market achieved a value of almost USD 58 billion in 2022 and is projected to surpass USD 85 billion by 2027. The global *lactobacillus* probiotics market had a valuation of USD 1162.6 million in 2020. According to projections, the value is expected to reach USD 2529.5 million by 2032, with a compound annual growth rate (CAGR) of 6.6%.^129,130^ The discovery of powerful probiotic strains, as described by Kerry et al. that has been highlighted in our review, is expected to support the continuous enhancement of human health, opening the door for a significant rise in the market value soon.^5^ Currently up to the end of 2023 around 306 studies has been completed or recruiting (some with unknown status or terminated) with term “*lactobacillus* probiotic” according to the ClinicalTrials.gov. Dronkers et al. also give a brief review up to August 1, 2019 about the clinical studies of probiotic, in which *L. rhamnosus* GG (LGG) and *Bifidobacterium animalis ssp*. lactis BB12 are the probiotic strains studied most.^131^

However, overcoming substantial obstacles will be necessary to get positive outcomes in this scenario. Probiotic bacteria have been found to die due to many factors such as low pH during fermentation in food products, oxygen exposure during refrigeration, distribution, and storage, and acidic conditions in the human stomach.^132,133^ Regarding probiotic meals, there are extra problems related to sensory acceptability. Several studies indicate that when compared to traditional competitors, probiotic-enhanced products may be able to achieve comparable, if not higher, performance. Examples are *L. reuteri* RC-14 and *L. rhamnosus* GR-1 fortified functional yogurt, *L. paracasei* and inulin-enriched chocolate mousse, curdled milk enhanced with *L. acidophilus* and inulin, and inulin-supplemented milk fermentation with *B. animalis* and *L. acidophilus* La-5.^134–136^ Notably, in some cases major medical regulatory authorities like the European Food Safety Authority and the US Food and Drug Administration have yet to endorse any probiotic formulation as a therapeutic intervention. Consequently, the marketing of probiotics as dietary supplements tends to emphasize characteristics such as safety, viability in the gastrointestinal tract, and minimal impact on food taste, rather than unequivocal health-promoting effects.^137^ As dietary therapies that aim to modify the gut microbiota may hold therapeutic potential in treating disorders, given the significant role the gut microbiota plays in maintaining host health. A community-specific consensus is necessary to develop customized nutritional recommendations, effectively implement the microbiome-based modulation strategy, and accurately evaluate its effectiveness.^9^

Probiotics are important for maintaining gastrointestinal health, boosting immune system function, and promoting metabolic balance. When taken correctly, they can offer advantages such as decreasing the likelihood of gastrointestinal illnesses, boosting immunity, and promoting metabolic health. Understanding the intricacies of the microbiota and the impact of probiotic microbes on health is a difficult task. Thoroughly planned, comprehensive research efforts that incorporate blinding and randomization, and are preferably free from the influence of commercial interests, are crucial for generating facts that will substantiate policy decisions. Objective examination of the data and stratification of the results are crucial in order to account for interindividual variables that may mislead or complicate the desired outcomes. Greater emphasis should be placed on the discovery, dissemination, and publication of data pertaining to adverse responses. Furthermore, providing a comprehensive elucidation of the mechanisms underlying the beneficial effects of probiotics in particular demographic subgroups has the capacity to enhance predictive precision, refine clinical trial methodologies, and advance the creation of strategies for probiotic health. The highlighted strains in this context exhibit enhanced probiotic characteristics, indicating potential future health benefits. In order to completely understand these *lactobacilli* and ultimately understand their possible health implications, it is imperative to meticulously evaluate the existing challenges.

## List of Abbreviations

*L. plantarum*: *Lactobacillus plantarum*
*L. paracasei*: *Lactobacillus paracasei*
*L. acidophilus*: *Lactobacillus acidophilus*
*L. casei*: *Lactobacillus casei*
*L. rhamnosus*: *Lactobacillus rhamnosus*
*L crispatus*: *Lactobacillus crispatus*
*L. gasseri*: *Lactobacillus gasseri*
*L. reuteri*: *Lactobacillus reuteri*
*L. bulgaricus*: *Lactobacillus bulgaricus*
LAB: Lactic acid bacteria
AIDS: Acquired Immune Deficiency Syndrome
GRAS: Generally regarded as safe
FAO: Food and Agriculture Organization
WHO: World Health Organization
FDA: Food and Drug Administration
GIT: Gastrointestinal tract
LP-PBF: *L. paracasei* CNCM I-5220-derived postbiotic
qPCR: Quantitative polymerase chain reaction
DAF: Decay accelerating factor
CFU: Colony forming units
TLR4: Toll-like receptor 4
BSSL: Bile salt – stimulated lipase
PTL: Pancreatic triglyceride lipas
PLRP2: Pancreatic lipase-related protein 2
*L. zeae*: *Lactobacillus zeae*
Th17: T helper type 17
Treg: Regulatory T
NF- κB: Nuclear factor-kappa B
NFATc1: Nuclear factor of activated T-Cells 1
UTI: urinary tract infection
PLGA: Poly (lactic-co-glycolic acid)
PEO: Polyethylene oxide

## Data Availability

The relevant research articles were reviewed for this article.

## References

[1] Zheng J, Wittouck S, Salvetti E, Franz CMAP, Harris HMB, Mattarelli P, O’Toole PW, Pot B, Vandamme P, Walter J, et al. A taxonomic note on the genus lactobacillus: description of 23 novel genera, emended description of the genus lactobacillus beijerinck 1901, and union of lactobacillaceae and leuconostocaceae. Int J Systematic Evol Microbiol. 2020;70(4):2782–21. doi:10.1099/ijsem.0.004107. 32293557

[2] Jeong J-J, Park HJ, Cha MG, Park E, Won S-M, Ganesan R, Gupta H, Gebru YA, Sharma SP, Lee SB, et al. The lactobacillus as a probiotic: focusing on liver diseases. Microorganisms. 2022;10(2):288. doi:10.3390/microorganisms10020288. 35208742 PMC8879051

[3] Martinez RM, Hulten KG, Bui U, Clarridge JE, Onderdonk AB. Molecular analysis and clinical significance of lactobacillus spp. Recovered from clinical specimens presumptively associated with disease. J Clin Microbiol. 2014;52(1):30–36. doi:10.1128/JCM.02072-13. 24131686 PMC3911440

[4] Belkaid Y, Hand T. Role of the microbiota in immunity and inflammation. Cell. 2014;157(1):121–141. doi:10.1016/j.cell.2014.03.011. 24679531 PMC4056765

[5] Dempsey E, Corr SC. Lactobacillus spp. For gastrointestinal health: current and future perspectives. Front Immunol [Internet]. 2022 [accessed 2023 Nov 29]. 13. 10.3389/fimmu.2022.840245. PMC9019120 35464397

[6] Hammes WP, Hertel C. Lactobacillus [Internet]. In: Bergey’s manual of systematics of archaea and bacteria. John Wiley & Sons, Ltd; 2015 [accessed 2023 Nov 29]. p. 1–76. 10.1002/9781118960608.gbm00604.

[7] Mital BK, Garg SK. Anticarcinogenic, hypocholesterolemic, and antagonistic activities of Lactobacillus acidophilus. Crit Rev Microbiol. 1995;21(3):175–214. doi:10.3109/10408419509113540. 8845062

[8] Zhang T, Liu W, Lu H, Cheng T, Wang L, Wang G, Zhang H, Chen W. Lactic acid bacteria in relieving constipation: mechanism, clinical application, challenge, and opportunity. Crit Rev Food Sci Nutr. 2023;1–24. doi:10.1080/10408398.2023.2278155. 37971876

[9] Ma T, Shen X, Shi X, Sakandar HA, Quan K, Li Y, Jin H, Kwok L-Y, Zhang H, Sun Z. Targeting gut microbiota and metabolism as the major probiotic mechanism - an evidence-based review. Trends Food Sci Technol. 2023;138:178–198. doi:10.1016/j.tifs.2023.06.013.

[10] Kerry RG, Patra JK, Gouda S, Park Y, Shin H-S, Das G. Benefaction of probiotics for human health: a review. J Food Drug Anal. 2018;26(3):927–939. doi:10.1016/j.jfda.2018.01.002. 29976412 PMC9303019

[11] Pandey Kavita R, Naik Suresh R, Vakil Babu V. Probiotics, prebiotics and synbiotics- a review. J Food Sci Technol. 2015;52(12):7577–7587. doi:10.1007/s13197-015-1921-1. 26604335 PMC4648921

[12] Fijan S. Microorganisms with claimed probiotic properties: an overview of recent literature. Int J Environ Res Public Health. 2014;11(5):4745–4767. doi:10.3390/ijerph110504745. 24859749 PMC4053917

[13] Behera SS, Ray RC, Zdolec N. Lactobacillus plantarum with functional properties: an approach to increase safety and shelf-life of fermented foods. Biomed Res Int. 2018;2018:1–18. doi:10.1155/2018/9361614. PMC5994577 29998137

[14] Jin YJ, Park YK, Cho MS, Lee ES, Park DS. New insight and metrics to understand the ontogeny and succession of lactobacillus plantarum subsp. plantarum and lactobacillus plantarum subsp. argentoratensis. Sci Rep. 2018;8(1):6029. doi:10.1038/s41598-018-24541-6. 29662105 PMC5902611

[15] Todorov SD, Franco BDGDM. Lactobacillus plantarum: characterization of the species and application in food production. Food Rev Int. 2010;26(3):205–229. doi:10.1080/87559129.2010.484113.

[16] Capozzi V, Russo P, Ladero V, Fernandez M, Fiocco D, Alvarez MA, Grieco F, Spano G. Biogenic amines degradation by lactobacillus plantarum: toward a potential application in wine. Front Microbiol [Internet]. 2012 [accessed 2023 Nov 29]. 3. 10.3389/fmicb.2012.00122. PMC3316997 22485114

[17] Russo P, Arena MP, Fiocco D, Capozzi V, Drider D, Spano G. Lactobacillus plantarum with broad antifungal activity: a promising approach to increase safety and shelf-life of cereal-based products. Int J Food Microbiol. 2017;247:48–54. doi:10.1016/j.ijfoodmicro.2016.04.027. 27240933

[18] Lee J-J, Kwon H, Lee J-H, Kim D-G, Jung S-H, Ma JY. Fermented soshiho-tang with lactobacillus plantarum enhances the antiproliferative activity in vascular smooth muscle cell. BMC Complement Alternative Med. 2014;14(1):78. doi:10.1186/1472-6882-14-78. PMC3942327 24580756

[19] Zhang Q, Zhao Q, Li T, Lu L, Wang F, Zhang H, Liu Z, Ma H, Zhu Q, Wang J, et al. Lactobacillus plantarum-derived indole-3-lactic acid ameliorates colorectal tumorigenesis via epigenetic regulation of CD8+ T cell immunity. Cell Metab. 2023;35(6):943–960.e9. doi:10.1016/j.cmet.2023.04.015. 37192617

[20] Zhu R, Fang Y, Li H, Liu Y, Wei J, Zhang S, Wang L, Fan R, Wang L, Li S, et al. Psychobiotic lactobacillus plantarum JYLP-326 relieves anxiety, depression, and insomnia symptoms in test anxious college via modulating the gut microbiota and its metabolism. Front Immunol. 2023;14:1158137. doi:10.3389/fimmu.2023.1158137. 37033942 PMC10077425

[21] Wang L, Zhao Z, Zhao L, Zhao Y, Yang G, Wang C, Gao L, Niu C, Li S. Lactobacillus plantarum DP189 reduces α-syn aggravation in MPTP-Induced Parkinson’s disease mice via regulating oxidative damage, inflammation, and gut microbiota disorder. J Agric Food Chem. 2022;70(4):1163–1173. doi:10.1021/acs.jafc.1c07711. 35067061

[22] Mo S-J, Lee K, Hong H-J, Hong D-K, Jung S-H, Park S-D, Shim J-J, Lee J-L. Effects of lactobacillus curvatus HY7601 and lactobacillus plantarum KY1032 on overweight and the gut microbiota in humans: randomized, double-blinded, placebo-controlled clinical trial. Nutrients. 2022;14(12):2484. doi:10.3390/nu14122484. 35745214 PMC9228474

[23] Liu N, Miao S, Qin L. Screening and application of lactic acid bacteria and yeasts with l-lactic acid-producing and antioxidant capacity in traditional fermented rice acid. Food Sci Nutr. 2020;8(11):6095–6111. doi:10.1002/fsn3.1900. 33282261 PMC7684631

[24] Smokvina T, Wels M, Polka J, Chervaux C, Brisse S, Boekhorst J, Vlieg JET, van H, Siezen RJ, Highlander SK. Lactobacillus paracasei comparative genomics: towards species pan-genome definition and exploitation of diversity. PLoS One. 2013;8(7):e68731. doi:10.1371/journal.pone.0068731. 23894338 PMC3716772

[25] Sornsenee P, Chimplee S, Saengsuwan P, Romyasamit C. Characterization of probiotic properties and development of banana powder enriched with freeze-dried Lacticaseibacillus paracasei probiotics. Heliyon. 2022;8(10):e11063. doi:10.1016/j.heliyon.2022.e11063. 36276732 PMC9578979

[26] Wang MF, Lin HC, Wang YY, Hsu CH. Treatment of perennial allergic rhinitis with lactic acid bacteria. Pediatr Allergy Immunol. 2004;15(2):152–158. doi:10.1111/j.1399-3038.2004.00156.x. 15059192

[27] Perrin Y, Nutten S, Audran R, Berger B, Bibiloni R, Wassenberg J, Barbier N, Aubert V, Moulin J, Singh A, et al. Comparison of two oral probiotic preparations in a randomized crossover trial highlights a potentially beneficial effect of lactobacillus paracasei NCC2461 in patients with allergic rhinitis. Clin Transl Allergy. 2014;4(1):1. doi:10.1186/2045-7022-4-1. 24393277 PMC3925289

[28] Terpou A, Mantzourani I, Galanis A, Kanellaki M, Bezirtzoglou E, Bekatorou A, Koutinas AA, Plessas S. Employment of L. paracasei K5 as a novel potentially probiotic freeze-dried Starter for feta-type cheese production. Microorganisms. 2019;7(1):3. doi:10.3390/microorganisms7010003. PMC6352075 30587786

[29] Algieri F, Tanaskovic N, Rincon CC, Notario E, Braga D, Pesole G, Rusconi R, Penna G, Rescigno M. Lactobacillus paracasei CNCM I-5220-derived postbiotic protects from the leaky-gut. Front Microbiol. 2023;14:1157164. doi:10.3389/fmicb.2023.1157164. 37020718 PMC10067918

[30] Kumaree KK, Prasanth MI, Sivamaruthi BS, Kesika P, Tencomnao T, Chaiyasut C, Prasansuklab A. Lactobacillus paracasei HII01 enhances lifespan and promotes neuroprotection in caenorhabditis elegans. Sci Rep. 2023;13(1):16707. doi:10.1038/s41598-023-43846-9. 37794096 PMC10550917

[31] Huang Z, Zhou X, Stanton C, Ross RP, Zhao J, Zhang H, Yang B, Chen W. Comparative genomics and specific functional characteristics analysis of Lactobacillus acidophilus. Microorganisms. 2021;9:1992. 34576887 10.3390/microorganisms9091992 PMC8464880

[32] Crawley AB, Barrangou R. Conserved genome organization and core transcriptome of the Lactobacillus acidophilus complex. Front Microbiol [Internet] 2018 [accessed 2024 Jul 23]. 9. https://www.frontiersin.org/journals/microbiology/articles/10.3389/fmicb.2018.01834/full. 10.3389/fmicb.2018.01834 PMC6099100 30150974

[33] Gao H, Li X, Chen X, Hai D, Wei C, Zhang L, Li P. The functional roles of Lactobacillus acidophilus in different physiological and pathological processes. J Microbiol Biotechnol. 2022;32(10):1226–1233. doi:10.4014/jmb.2205.05041. 36196014 PMC9668099

[34] Gilliland SE, Speck ML. Antagonistic action of Lactobacillus acidophilus toward intestinal and foodborne pathogens in associative Cultures1. J Food Protect. 1977;40(12):820–823. doi:10.4315/0362-028X-40.12.820. 30736216

[35] Farahmand N, Ouoba LII, Naghizadeh Raeisi S, Sutherland J, Ghoddusi HB. Probiotic lactobacilli in fermented dairy products: selective detection, enumeration and identification scheme. Microorganisms. 2021;9(8):1600. doi:10.3390/microorganisms9081600. 34442679 PMC8401870

[36] Harrison VC, Peat G. Serum cholesterol and bowel flora in the newborn. Am J Clin Nutr. 1975;28(12):1351–1355. doi:10.1093/ajcn/28.12.1351. 45573

[37] Goldin BR, Gorbach SL. The effect of milk and lactobacillus feeding on human intestinal bacterial enzyme activity. Am J Clin Nutr. 1984;39(5):756–761. doi:10.1093/ajcn/39.5.756. 6424430

[38] Zhao R, Sun J, Mo H, Zhu Y. Analysis of functional properties of Lactobacillus acidophilus. World J Microbiol Biotechnol. 2007;23(2):195–200. doi:10.1007/s11274-006-9209-2.

[39] Jeon S, Hwang J, Do H, Le LTHL, Lee CW, Yoo W, Lee MJ, Shin SC, Kim KK, Kim H-W, et al. Feruloyl esterase (LaFae) from Lactobacillus acidophilus: structural insights and functional characterization for application in ferulic acid production. Int J Mol Sci. 2023;24(13):11170. doi:10.3390/ijms241311170. 37446348 PMC10342849

[40] Moshiri M, Dallal MMS, Rezaei F, Douraghi M, Sharifi L, Noroozbabaei Z, Gholami M, Mirshafiey A. The effect of Lactobacillus acidophilus PTCC 1643 on cultured intestinal epithelial cells infected with Salmonella enterica serovar enteritidis. Osong Public Health Res Perspect. 2017;8(1):54–60. doi:10.24171/j.phrp.2017.8.1.07. 28443224 PMC5402851

[41] Cataruci ACS, Kawamoto D, Shimabukuro N, Ishikawa KH, Ando-Suguimoto ES, Ribeiro RA, Nicastro GG, Albuquerque-Souza E, de Souza RF, Mayer MPA. Oral administration of Lactobacillus acidophilus LA5 prevents alveolar bone loss and alters oral and gut microbiomes in a murine periodontitis experimental Model. Microorganisms. 2024;12(6):1057. doi:10.3390/microorganisms12061057. 38930439 PMC11205731

[42] Banks JM, Williams AG. The role of the nonstarter lactic acid bacteria in cheddar cheese ripening. Int J Dairy Technol. 2004;57(2–3):145–152. doi:10.1111/j.1471-0307.2004.00150.x.

[43] Randazzo CL, Restuccia C, Romano AD, Caggia C. Lactobacillus casei, dominant species in naturally fermented Sicilian green olives. Int J Food Microbiol. 2004;90(1):9–14. doi:10.1016/S0168-1605(03)00159-4. 14672826

[44] McFarland LV. Evidence-based review of probiotics for antibiotic-associated diarrhea and Clostridium difficile infections. Anaerobe. 2009;15(6):274–280. doi:10.1016/j.anaerobe.2009.09.002. 19825425

[45] Zhu H, Cao C, Wu Z, Zhang H, Sun Z, Wang M, Xu H, Zhao Z, Wang Y, Pei G, et al. The probiotic L. casei Zhang slows the progression of acute and chronic kidney disease. Cell Metab. 2021;33(10):1926–1942.e8. doi:10.1016/j.cmet.2021.06.014. 34270930

[46] Favero C, Ortiz A, Sanchez-Niño MD. Probiotics for kidney disease. Clin Kidney J. 2022;15(11):1981–1986. doi:10.1093/ckj/sfac056. 36325000 PMC9613434

[47] Zhu Z, Yi B, Tang Z, Chen X, Li M, Xu T, Zhao Z, Tang C. Lactobacillus casei combined with lactobacillus reuteri alleviate pancreatic cancer by inhibiting TLR4 to promote macrophage M1 polarization and regulate gut microbial homeostasis. BMC Cancer. 2023;23(1):1044. doi:10.1186/s12885-023-11557-z. 37904102 PMC10614400

[48] Hosseinzadeh R, Bahadori A, Ghorbani M, Mohammadimehr M. Lactobacillus casei condition medium downregulates miR-21 relative expression in HT-29 colorectal cancer cell line. FEMS Microbiol Lett. 2023;370:fnad089. doi:10.1093/femsle/fnad089. 37697675

[49] Saidi N, Saderi H, Owlia P, Soleimani M. Anti-biofilm potential of lactobacillus casei and lactobacillus rhamnosus cell-free supernatant extracts against staphylococcus aureus. Adv Biomed Res. 2023;12(1):50. doi:10.4103/abr.abr_156_21. 37057221 PMC10086653

[50] Hill D, Sugrue I, Tobin C, Hill C, Stanton C, Ross RP. The Lactobacillus casei group: history and health related applications. Front Microbiol [Internet] 2018 [accessed 2024 Sep 21]. 9. https://www.frontiersin.org/journals/microbiology/articles/10.3389/fmicb.2018.02107/full. 10.3389/fmicb.2018.02107 PMC6160870 30298055

[51] Pimentel TC, Brandão LR, de Oliveira MP, da Costa WKA, Magnani M. Health benefits and technological effects of Lacticaseibacillus casei-01: an overview of the scientific literature. Trends Food Sci Technol. 2021;114:722–737. doi:10.1016/j.tifs.2021.06.030.

[52] Wuyts S, Wittouck S, De Boeck I, Allonsius CN, Pasolli E, Segata N, Lebeer S, Dorrestein PC. Large-scale phylogenomics of the Lactobacillus casei group highlights taxonomic inconsistencies and reveals novel clade-associated features. mSystems. 2017;2(4):10.1128/msystems.00061-17. doi:10.1128/msystems.00061-17. PMC5566788 28845461

[53] Liu DD, Gu CT. Proposal to reclassify Lactobacillus zhaodongensis, Lactobacillus zeae, Lactobacillus argentoratensis and Lactobacillus buchneri subsp. silagei as lacticaseibacillus zhaodongensis comb. nov. lacticaseibacillus zeae comb. nov. lactiplantibacillus argentoratensis comb. nov. And Lentilactobacillus buchneri subsp. silagei comb. nov. respectively and apilactobacillus kosoi as a later heterotypic synonym of Apilactobacillus micheneri. Int J Systematic Evol Microbiol. 2020;70(12):6414–6417. doi:10.1099/ijsem.0.004548. 33112225

[54] Österlund P, Ruotsalainen T, Korpela R, Saxelin M, Ollus A, Valta P, Kouri M, Elomaa I, Joensuu H. Lactobacillus supplementation for diarrhoea related to chemotherapy of colorectal cancer: a randomised study. Br J Cancer. 2007;97(8):1028–1034. doi:10.1038/sj.bjc.6603990. 17895895 PMC2360429

[55] Guandalini S, Pensabene L, Zikri MA, Dias JA, Casali LG, Hoekstra H, Kolacek S, Massar K, Micetic–Turk D, Papadopoulou A, et al. Lactobacillus GG administered in oral rehydration solution to children with acute diarrhea: a multicenter European trial. J Pediatr Gastroenterol Nutr. 2000;30(1):54. doi:10.1097/00005176-200001000-00018. 10630440

[56] Hojsak I, Fabiano V, Pop TL, Goulet O, Zuccotti GV, Çokuğraş FC, Pettoello-Mantovani M, Kolaček S. Guidance on the use of probiotics in clinical practice in children with selected clinical conditions and in specific vulnerable groups. Acta Paediatrica. 2018;107(6):927–937. doi:10.1111/apa.14270. 29446865 PMC5969308

[57] Cameron D, Hock QS, Kadim M, Mohan N, Ryoo E, Sandhu B, Yamashiro Y, Jie C, Hoekstra H, Guarino A. Probiotics for gastrointestinal disorders: proposed recommendations for children of the Asia-Pacific region. World J Gastroenterol. 2017;23(45):7952–7964. doi:10.3748/wjg.v23.i45.7952. 29259371 PMC5725290

[58] Blaabjerg S, Artzi DM, Aabenhus R. Probiotics for the prevention of antibiotic-associated diarrhea in outpatients—A systematic review and meta-analysis. Antibiotics. 2017;6(4):21. doi:10.3390/antibiotics6040021. 29023420 PMC5745464

[59] Mathipa-Mdakane MG, Thantsha MS. Lacticaseibacillus rhamnosus: a suitable Candidate for the construction of novel bioengineered probiotic strains for targeted pathogen control. Foods. 2022;11(6):785. doi:10.3390/foods11060785. 35327208 PMC8947445

[60] Guo M, Liu H, Yu Y, Zhu X, Xie H, Wei C, Mei C, Shi Y, Zhou N, Qin K, et al. Lactobacillus rhamnosus GG ameliorates osteoporosis in ovariectomized rats by regulating the Th17/Treg balance and gut microbiota structure. Gut Microbes. 2023;15(1):2190304. doi:10.1080/19490976.2023.2190304. 36941563 PMC10038048

[61] Fu J, Jia L, Wu L, Jiang Y, Zhao R, Du J, Guo L, Zhang C, Xu J, Liu Y. Lactobacillus rhamnosus inhibits osteoclast differentiation by suppressing the TLR2/NF-κB pathway. Oral Dis. 2023;30(4):2373–2386. doi:10.1111/odi.14712. 37602540

[62] Tripathy A, Swain N, Padhan P, Raghav SK, Gupta B. Lactobacillus rhamnosus reduces CD8+T cell mediated inflammation in patients with rheumatoid arthritis. Immunobiology. 2023;228(4):152415. doi:10.1016/j.imbio.2023.152415. 37356231

[63] Ojala T, Kuparinen V, Koskinen JP, Alatalo E, Holm L, Auvinen P, Edelman S, Westerlund-Wikström B, Korhonen TK, Paulin L, et al. Genome sequence of lactobacillus crispatus ST1. J Bacteriol. 2010;192(13):3547–3548. doi:10.1128/JB.00399-10. 20435723 PMC2897677

[64] Donnarumma G, Molinaro A, Cimini D, De Castro C, Valli V, De Gregorio V, De Rosa M, Schiraldi C. Lactobacillus crispatus L1: high cell density cultivation and exopolysaccharide structure characterization to highlight potentially beneficial effects against vaginal pathogens. BMC Microbiol. 2014;14(1):137. doi:10.1186/1471-2180-14-137. 24884965 PMC4054921

[65] Czaja CA, Stapleton AE, Yarova-Yarovaya Y, Stamm WE. Phase I trial of a lactobacillus crispatus vaginal suppository for prevention of recurrent urinary tract infection in women. Infect Dis Obstet Gynecol. 2007;2007:1–8. doi:10.1155/2007/35387. PMC2216064 18288237

[66] Dwyer JP, Dwyer PL. Lactobacillus probiotics may prevent recurrent UTIs in postmenopausal women. BMJ Evidence-Based Med. 2013;18(4):141–142. doi:10.1136/eb-2012-100961. 23125237

[67] Antonio MAD, Meyn LA, Murray PJ, Busse B, Hillier SL. Vaginal colonization by Probiotic Lactobacillus crispatus CTV-05 is decreased by sexual activity and endogenous lactobacilli. J Infect Dis. 2009;199(10):1506–1513. doi:10.1086/598686. 19331578

[68] Vásquez A, Jakobsson T, Ahrné S, Forsum U, Molin G. Vaginal lactobacillus flora of healthy Swedish women. J Clin Microbiol. 2002;40(8):2746–2749. doi:10.1128/JCM.40.8.2746-2749.2002. 12149323 PMC120688

[69] Mahmoud MY, Wesley M, Kyser A, Lewis WG, Lewis AL, Steinbach-Rankins JM, Frieboes HB. Lactobacillus crispatus-loaded electrospun fibers yield viable and metabolically active bacteria that kill gardnerella in vitro. Eur J Pharm Biopharm. 2023;187:68–75. doi:10.1016/j.ejpb.2023.04.011. 37086869 PMC10192109

[70] Wu F, Kong Y, Chen W, Liang D, Xiao Q, Hu L, Tan X, Wei J, Liu Y, Deng X, et al. Improvement of vaginal probiotics lactobacillus crispatus on intrauterine adhesion in mice model and in clinical practice. BMC Microbiol. 2023;23(1):78. doi:10.1186/s12866-023-02823-y. 36949381 PMC10032012

[71] Wu J-J, Zhou Q-Y, Liu D-M, Xiong J, Liang M-H, Tang J, Xu Y-Q. Evaluation of the safety and probiotic properties of Lactobacillus gasseri LGZ1029 based on whole genome analysis. LWT. 2023;184:114759. doi:10.1016/j.lwt.2023.114759.

[72] Selle K, Klaenhammer TR. Genomic and phenotypic evidence for probiotic influences of Lactobacillus gasseri on human health. FEMS Microbiol Rev. 2013;37(6):915–935. doi:10.1111/1574-6976.12021. 23488471

[73] Yamada N, Iwamoto C, Kano H, Yamaoka N, Fukuuchi T, Kaneko K, Asami Y. Evaluation of purine utilization by Lactobacillus gasseri strains with potential to decrease the absorption of food-derived purines in the human intestine. Nucleosides Nucleotides Nucleic Acids. 2016;35(10–12):670–676. doi:10.1080/15257770.2015.1125000. 27906630

[74] Mann S, Park MS, Johnston TV, Ji GE, Hwang KT, Ku S. Oral probiotic activities and biosafety of Lactobacillus gasseri HHuMIN D. Microb Cell Fact. 2021;20(1):75. doi:10.1186/s12934-021-01563-w. 33757506 PMC7986493

[75] Cotter PD, Ross RP, Hill C. Bacteriocins — a viable alternative to antibiotics? Nat Rev Microbiol. 2013;11(2):95–105. doi:10.1038/nrmicro2937. 23268227

[76] Pandey N, Malik RK, Kaushik JK, Singroha G. Gassericin A: a circular bacteriocin produced by lactic acid bacteria Lactobacillus gasseri. World J Microbiol Biotechnol. 2013;29(11):1977–1987. doi:10.1007/s11274-013-1368-3. 23712477

[77] Hu J, Hou Q, Zheng W, Yang T, Yan X. Lactobacillus gasseri LA39 promotes hepatic primary bile acid biosynthesis and intestinal secondary bile acid biotransformation. J Zhejiang Univ Sci B. 2023;24:734–748. 37551559 10.1631/jzus.B2200439 PMC10423968

[78] Gao Q, Fan T, Luo S, Zheng J, Zhang L, Cao L, Zhang Z, Li L, Huang Z, Zhang H, et al. Lactobacillus gasseri LGV03 isolated from the cervico-vagina of hpv-cleared women modulates epithelial innate immune responses and suppresses the growth of hpv-positive human cervical cancer cells. Transl Oncol. 2023;35:101714. doi:10.1016/j.tranon.2023.101714. 37331103 PMC10366645

[79] Jiang S, Liu A, Ma W, Liu X, Luo P, Zhan M, Zhou X, Chen L, Zhang J. Lactobacillus gasseri CKCC1913 mediated modulation of the gut–liver axis alleviated insulin resistance and liver damage induced by type 2 diabetes. Food Funct. 2023;14(18):8504–8520. doi:10.1039/D3FO01701J. 37655696

[80] Mu Q, Tavella VJ, Luo XM. Role of Lactobacillus reuteri in human health and diseases. Front Microbiol. 2018;9:757. doi:10.3389/fmicb.2018.00757. 29725324 PMC5917019

[81] Ruiz-Palacios G, Guerrero ML, Hilty M, Dohnalek M, Newton P, Calva JJ, Costigan T, Tuz F, Arteaga F. Feeding of a Probiotic for the prevention of community-acquired diarrhea in young Mexican children. † 1089. Pediatr Res. 1996;39:184–184. doi:10.1203/00006450-199604001-01111.

[82] Mao Y, Nobaek S, Kasravi B, Adawi D, Stenram U, Molin G, Jeppsson B. The effects of lactobacillus strains and oat fiber on methotrexate- induced enterocolitis in rats. Gastroenterology. 1996;111(2):334–344. doi:10.1053/gast.1996.v111.pm8690198. 8690198

[83] Nikawa H, Makihira S, Fukushima H, Nishimura H, Ozaki Y, Ishida K, Darmawan S, Hamada T, Hara K, Matsumoto A, et al. Lactobacillus reuteri in bovine milk fermented decreases the oral carriage of mutans streptococci. Int J Food Microbiol. 2004;95(2):219–223. doi:10.1016/j.ijfoodmicro.2004.03.006. 15282133

[84] Bender MJ, McPherson AC, Phelps CM, Pandey SP, Laughlin CR, Shapira JH, Medina Sanchez L, Rana M, Richie TG, Mims TS, et al. Dietary tryptophan metabolite released by intratumoral lactobacillus reuteri facilitates immune checkpoint inhibitor treatment. Cell. 2023;186(9):1846–1862.e26. doi:10.1016/j.cell.2023.03.011. 37028428 PMC10148916

[85] Shen J, Wang S, Huang Y, Wu Z, Han S, Xia H, Chen H, Li L. Lactobacillus reuteri ameliorates lipopolysaccharide-induced acute lung injury by modulating the gut microbiota in mice. Nutrients. 2023;15(19):4256. doi:10.3390/nu15194256. 37836540 PMC10574429

[86] Hao P, Zheng H, Yu Y, Ding G, Gu W, Chen S, Yu Z, Ren S, Oda M, Konno T, et al. Complete sequencing and Pan-genomic analysis of Lactobacillus delbrueckii subsp. bulgaricus reveal its genetic basis for industrial yogurt production. PLoS One. 2011;6(1):e15964. doi:10.1371/journal.pone.0015964. 21264216 PMC3022021

[87] Lorenzo JM, Munekata PE, Dominguez R, Pateiro M, Saraiva JA, Franco D. Chapter 3 - main groups of microorganisms of relevance for food safety and stability: general aspects and overall description [internet]. In: Barba F, Sant’Ana A, Orlien V Koubaa M, editors. Innovative technologies for food preservation. Academic Press; 2018 [accessed 2024 Sep 22]. p. 53–107. https://www.sciencedirect.com/science/article/pii/B9780128110317000030.

[88] Silveira DSC, Veronez LC, Lopes-Júnior LC, Anatriello E, Brunaldi MO, Pereira-da-Silva G. Lactobacillus bulgaricus inhibits colitis-associated cancer via a negative regulation of intestinal inflammation in azoxymethane/dextran sodium sulfate model. World J Gastroenterol. 2020;26(43):6782–6794. doi:10.3748/wjg.v26.i43.6782. 33268961 PMC7684459

[89] Shi L-E, Li Z-H, Li D-T, Xu M, Chen H-Y, Zhang Z-L, Tang Z-X. Encapsulation of probiotic lactobacillus bulgaricus in alginate–milk microspheres and evaluation of the survival in simulated gastrointestinal conditions. J Food Eng. 2013;117(1):99–104. doi:10.1016/j.jfoodeng.2013.02.012.

[90] Remaggi G, Bottari B, Bancalari E, Catanzano O, Neviani E, Elviri L. Lactobacillus delbrueckii subsp. bulgaricus derivatives for 3D printed alginate/hyaluronic acid self-crosslinking hydrogels: manufacturing and wound healing potential. Int J Biol Macromol. 2023;242:124454. doi:10.1016/j.ijbiomac.2023.124454. 37076070

[91] Xue ZP, Cu X, Xu K, Peng JH, Liu HR, Zhao RT, Wang Z, Wang T, Xu ZS. The effect of glutathione biosynthesis of streptococcus thermophilus ST-1 on cocultured Lactobacillus delbrueckii ssp. bulgaricus ATCC11842. J Dairy Sci. 2023;106(2):884–896. doi:10.3168/jds.2022-22123. 36460506

[92] Guo Y, Zhao Y, Gao Y, Wang G, Zhao Y, Zhang J, Li Y, Wang X, Liu J, Chen G. Low acyl gellan gum immobilized lactobacillus bulgaricus T15 produce D-lactic acid from non-detoxified corn stover hydrolysate. Biotechnol Biofuels Bioprod. 2023;16(1):43. doi:10.1186/s13068-023-02292-5. 36915198 PMC10009946

[93] Leeuwendaal NK, Stanton C, O’Toole PW, Beresford TP. Fermented foods, health and the gut microbiome. Nutrients. 2022;14(7):1527. doi:10.3390/nu14071527. 35406140 PMC9003261

[94] Widyastuti Y, Febrisiantosa A, Tidona F. Health-promoting properties of lactobacilli in fermented dairy products. Front Microbiol [Internet]. 2021 [accessed 2023 Dec 1]. 12. 10.3389/fmicb.2021.673890. PMC8175972 34093496

[95] Adolfsson O, Meydani SN, Russell RM. Yogurt and gut function. Am J Clin Nutr. 2004;80(2):245–256. doi:10.1093/ajcn/80.2.245. 15277142

[96] Afzaal M, Saeed F, Anjum F, Waris N, Husaain M, Ikram A, Ateeq H, Muhammad Anjum F, Suleria H. Nutritional and ethnomedicinal scenario of koumiss: a concurrent review. Food Sci Nutr. 2021;9(11):6421–6428. doi:10.1002/fsn3.2595. 34760271 PMC8565204

[97] Azat R, Liu Y, Li W, Kayir A, Lin D, Zhou W, Zheng X. Probiotic properties of lactic acid bacteria isolated from traditionally fermented Xinjiang cheese. J Zhejiang Univ Sci B. 2016;17:597–609. 27487805 10.1631/jzus.B1500250 PMC4980438

[98] Zheng X, Shi X, Wang B. A review on the general cheese processing technology, flavor biochemical pathways and the influence of yeasts in cheese. Front Microbiol. 2021;12:703284. doi:10.3389/fmicb.2021.703284. 34394049 PMC8358398

[99] Slattery C, Cotter PD, O’Toole W. Analysis of health benefits conferred by lactobacillus species from Kefir. Nutrients. 2019;11(6):1252. doi:10.3390/nu11061252. 31159409 PMC6627492

[100] Zabat MA, Sano WH, Wurster JI, Cabral DJ, Belenky P. Microbial community analysis of sauerkraut fermentation reveals a stable and rapidly established community. Foods. 2018;7(5):77. doi:10.3390/foods7050077. 29757214 PMC5977097

[101] Hongu N, Kim AS, Suzuki A, Wilson H, Tsui KC, Park S. Korean kimchi: promoting healthy meals through cultural tradition. J Ethnic Foods. 2017;4(3):172–180. doi:10.1016/j.jef.2017.08.005.

[102] Ju KH, Eung-Soo H, Ju KH, Eung-Soo H. Health promoting effects of kimchi [internet]. [accessed 2023 Dec 1]. doi:10.4018/978-1-5225-0591-4.ch0041AD.

[103] Saeed F, Afzaal M, Shah YA, Khan MH, Hussain M, Ikram A, Ateeq H, Noman M, Saewan SA, Khashroum AO. Miso: a traditional nutritious & health‐endorsing fermented product. Food Sci Nutr. 2022;10(12):4103–4111. doi:10.1002/fsn3.3029. 36514754 PMC9731531

[104] Behera SS, El Sheikha AF, Hammami R, Kumar A. Traditionally fermented pickles: how the microbial diversity associated with their nutritional and health benefits? J Funct Foods. 2020;70:103971. doi:10.1016/j.jff.2020.103971.

[105] Gebreselassie N, Abrahamsen RK, Beyene F, Abay F, Narvhus JA. Chemical composition of naturally fermented buttermilk. Int J Dairy Technol. 2016;69(2):200–208. doi:10.1111/1471-0307.12236.

[106] Barus T, Giovania G, Lay BW. Lactic acid bacteria from tempeh and their ability to acidify soybeans in tempeh fermentation. Microbiol Indonesia. 2020;14(4):149–155. doi:10.5454/mi.14.4.4.

[107] Akamine IT, Mansoldo FRP, Vermelho AB. Probiotics in the sourdough bread fermentation: current status. Fermentation. 2023;9(2):90. doi:10.3390/fermentation9020090.

[108] de Oliveira Leite AM, Miguel MAL, Peixoto RS, Rosado AS, Silva JT, Paschoalin VMF. Microbiological, technological and therapeutic properties of kefir: a natural probiotic beverage. Braz J Microbiol. 2013;44(2):341–349. doi:10.1590/S1517-83822013000200001. 24294220 PMC3833126

[109] Plessas S, Nouska C, Mantzourani I, Kourkoutas Y, Alexopoulos A, Bezirtzoglou E. Microbiological exploration of different types of kefir grains. Fermentation. 2017;3(1):1. doi:10.3390/fermentation3010001.

[110] Ganatsios V, Nigam P, Plessas S, Terpou A. Kefir as a functional beverage gaining momentum towards its health promoting attributes. Beverages. 2021;7(3):48. doi:10.3390/beverages7030048.

[111] Bintsis T, Papademas P. The evolution of fermented milks, from artisanal to industrial products: a critical review. Fermentation. 2022;8(12):679. doi:10.3390/fermentation8120679.

[112] Garofalo C, Ferrocino I, Reale A, Sabbatini R, Milanović V, Alkić-Subašić M, Boscaino F, Aquilanti L, Pasquini M, Trombetta MF, et al. Study of kefir drinks produced by backslopping method using kefir grains from Bosnia and Herzegovina: microbial dynamics and volatilome profile. Food Res Int. 2020;137:109369. doi:10.1016/j.foodres.2020.109369. 33233071

[113] Chong AQ, Lau SW, Chin NL, Talib RA, Basha RK. Fermented beverage benefits: a comprehensive review and comparison of kombucha and kefir microbiome. Microorganisms. 2023;11(5):1344. doi:10.3390/microorganisms11051344. 37317318 PMC10221066

[114] Azizi NF, Kumar MR, Yeap SK, Abdullah JO, Khalid M, Omar AR, Osman Mohd A, Mortadza SAS, Alitheen NB. Kefir and its biological activities. Foods. 2021;10(6):1210. doi:10.3390/foods10061210. 34071977 PMC8226494

[115] Bourrie BCT, Willing BP, Cotter PD. The microbiota and health promoting characteristics of the fermented beverage kefir. Front Microbiol. 2016;7:647. doi:10.3389/fmicb.2016.00647. 27199969 PMC4854945

[116] Serafini F, Turroni F, Ruas-Madiedo P, Lugli GA, Milani C, Duranti S, Zamboni N, Bottacini F, van Sinderen D, Margolles A, et al. Kefir fermented milk and kefiran promote growth of bifidobacterium bifidum PRL2010 and modulate its gene expression. Int J Food Microbiol. 2014;178:50–59. doi:10.1016/j.ijfoodmicro.2014.02.024. 24667318

[117] Slover CM, Danziger L. Lactobacillus: a review. Clin Microbiol Newsl. 2008;30(4):23–27. doi:10.1016/j.clinmicnews.2008.01.006.

[118] Rossi F, Amadoro C, Colavita G. Members of the lactobacillus genus complex (LGC) as opportunistic pathogens: a review. Microorganisms [Internet]. 2019 [accessed 2023 Nov 29]. 7. https://www.ncbi.nlm.nih.gov/pmc/articles/PMC6560513/ 5):126. doi:10.3390/microorganisms7050126. 31083452 PMC6560513

[119] Sherid M, Samo S, Sulaiman S, Husein H, Sifuentes H, Sridhar S. Liver abscess and bacteremia caused by lactobacillus: role of probiotics? Case report and review of the literature. BMC Gastroenterol. 2016;16(1):138. doi:10.1186/s12876-016-0552-y. 27863462 PMC5116133

[120] Cannon JP, Lee TA, Bolanos JT, Danziger LH. Pathogenic relevance of Lactobacillus: a retrospective review of over 200 cases. Eur J Clin Microbiol Infect Dis. 2005;24(1):31–40. doi:10.1007/s10096-004-1253-y. 15599646

[121] Salvana EMT, Frank M. Lactobacillus endocarditis: case report and review of cases reported since 1992. J Infect. 2006;53(1):e5–10. doi:10.1016/j.jinf.2005.10.005. 16307799

[122] Cooper CD, Vincent A, Greene JN, Sandin RL, Cobian L. Lactobacillus bacteremia in febrile neutropenic patients in a cancer hospital. Clin Infect Dis. 1998;26(5):1247–1248. doi:10.1086/598365. 9597275

[123] Salminen MK, Rautelin H, Tynkkynen S, Poussa T, Saxelin M, Valtonen V, Järvinen A. Lactobacillus bacteremia, clinical significance, and patient outcome, with special focus on probiotic L. Rhamnosus GG. Clin Infect Dis. 2004;38(1):62–69. doi:10.1086/380455. 14679449

[124] Moradi Moghaddam O. Probiotics in critically Ill patients. Anesth Pain Med. 2011;1(2):58–60. doi:10.5812/aapm.2291. 25729656 PMC4335740

[125] Katkowska M, Garbacz K, Kusiak A. Probiotics: should all patients take them? Microorganisms. 2021;9(12):2620. doi:10.3390/microorganisms9122620. 34946221 PMC8706842

[126] Kothari D, Patel S, Kim S-K. Probiotic supplements might not be universally-effective and safe: a review. Biomed Pharmacother. 2019;111:537–547. doi:10.1016/j.biopha.2018.12.104. 30597307

[127] Butel M-J. Probiotics, gut microbiota and health. Médecineet Mal Infectieuses. 2014;44(1):1–8. doi:10.1016/j.medmal.2013.10.002. 24290962

[128] Saini R, Saini S, Sugandha S. Probiotics: the health boosters. J Cutan Aesthetic Surg. 2009;2(2):112. doi:10.4103/0974-2077.58530. PMC2918342 20808603

[129] Lactobacillus probiotics market size, share - forecast report 2032 [internet]. accessed 2024 Jul 23]; https://www.businessresearchinsights.com/market-reports/lactobacillus-probiotics-market-110151.

[130] Global probiotics estimated market value 2022-2027 [Internet]. Statista. [accessed 2024 Jul 23]; https://www.statista.com/statistics/821259/global-probioticsl-market-value/.

[131] Dronkers TMG, Ouwehand AC, Rijkers GT. Global analysis of clinical trials with probiotics. Heliyon. 2020;6(7):e04467. doi:10.1016/j.heliyon.2020.e04467. 32715136 PMC7371762

[132] Kailasapathy K, Chin J. Survival and therapeutic potential of probiotic organisms with reference to Lactobacillus acidophilus and bifidobacterium spp. Immunol Cell Biol. 2000;78(1):80–88. doi:10.1046/j.1440-1711.2000.00886.x. 10651933

[133] Evivie SE. Preliminary studies on pharmaceutical microencapsulation for synbiotic application. J Appl Nat Sci. 2013;5(2):488–496. doi:10.31018/jans.v5i2.358.

[134] Rodrigues D, Rocha-Santos TAP, Pereira CI, Gomes AM, Malcata FX, Freitas AC. The potential effect of FOS and inulin upon probiotic bacterium performance in curdled milk matrices. LWT Food Sci Technol. 2011;44(1):100–108. doi:10.1016/j.lwt.2010.05.021.

[135] Hekmat S, Reid G. Sensory properties of probiotic yogurt is comparable to standard yogurt. nutr Res. 2006;26(4):163–166. doi:10.1016/j.nutres.2006.04.004.

[136] Zommiti M, Feuilloley MGJ, Connil N. Update of probiotics in human world: a nonstop source of benefactions till the end of Time. Microorganisms. 2020;8(12):1907. doi:10.3390/microorganisms8121907. 33266303 PMC7760123

[137] Suez J, Zmora N, Segal E, Elinav E. The pros, cons, and many unknowns of probiotics. Nat Med. 2019;25(5):716–729. doi:10.1038/s41591-019-0439-x. 31061539

